# The Use of Sugarcane Bagasse to Remove the Organic Dyes from Wastewater

**DOI:** 10.1155/2021/5570806

**Published:** 2021-06-28

**Authors:** Meryem Kerrou, Najia Bouslamti, Abdelaziz Raada, Abdellah Elanssari, Driss Mrani, My Sliman Slimani

**Affiliations:** University Moulay Ismail of Meknes, Faculty of Sciences and Techniques Errachidia, Department of Chemistry, Laboratory of Chemistry, Environment, and Materials Analysis Team, Meknes, Morocco

## Abstract

In the present study, the potential of sugarcane bagasse (SCB) was evaluated by methylene blue (MB) retention. The selected low-cost adsorbent was characterized by scanning electron microscopy (SEM) coupled with energy-dispersive X-ray spectroscopy (EDX), Fourier transform infrared spectroscopy (FTIR), BET method, and determination of the point of zero charge (pHzpc). Batch kinetic and isothermal studies were performed to examine the effects of contact time, initial dye concentration, adsorbent dose, pH, and temperature. The results show that the kinetic study of MB adsorption on sugarcane bagasse is very fast; the equilibrium is reached after only 20 minutes. The kinetic model of pseudo-second-order and the Langmuir isotherm model perfectly explain the adsorption process of MB with a monolayer adsorption capacity equal to 49.261 mg·g^−1^ activation parameters' values such as free energy (Δ*G*°), enthalpy (Δ*H*°), and entropy (Δ*S*°) also determined as −4.35 kJ·mol^−1^, −31.062 kJ·mol^−1^, and −0.084 J·mol^−1^·K^−1^, respectively. Besides, the thermodynamic parameters of the methylene blue sugarcane bagasse system indicate that the exothermic adsorption process is spontaneous.

## 1. Introduction

**Figure 1:**
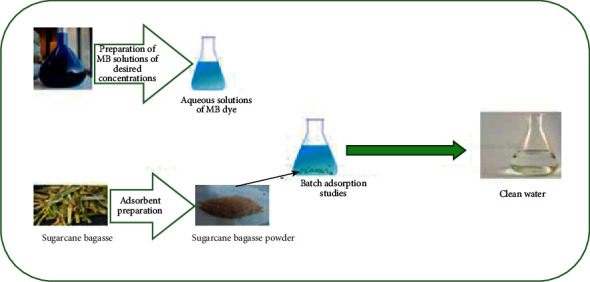
Adsorption process of methylene blue by sugarcane bagasse.

Industrial wastewater pollution has become a common problem in most countries [1] and one of the biggest environmental problems in recent decades. Industrial discharges include dyes used in different areas such as printing, food, cosmetics, and clinical products, but particularly in the textile industry [2]. The discharge of these colored effluents in nature affects not only humans and water but the whole environment. More than 14 million chemical products can be found in the environment; this number is gradually increasing which poses a negative impact on the biosphere and threats the balance of the natural ecosystem. Indeed, the toxic chemical products dumped into the water are genotoxic and mutagenic which might cause hereditary diseases that can be transmitted to the future generation [3]. Moreover, this will also provoke the increase of the chemical oxygen demand (COD) and biochemical oxygen demand (BOD) which inhibit and decrease the rate of photosynthesis and plants' growth [4]. In this context, a wide variety of physical, chemical, and biological techniques have been developed and tested in the treatment of these effluents loaded with dyes. Therefore, adsorption remains one of the easy-to-implement technologies, as it is widely used for water treatment [5]. Nowadays, adsorption has been accepted as a suitable removal technology, particularly for developing regions, because of its simple operation, the potential for regeneration. The adsorption process can be described as “the tendency of chemical species existing in a phase to adhere onto a solid” [6]. Technically, in adsorption science, the solid surface providing the adsorption sites is called adsorbent, and substances that are adsorbed at the solid surface are known as adsorbate [6]. The removal of dyes by adsorption on selective adsorbents such as low-cost adsorbents (sugarcane bagasse) as shown in Figure 1 has been synthesized and tested for dye removal.

From the literature, we can find that the use of activated carbon is considered a good adsorbent due to its high adsorption capacity for organic materials. However, activated carbon is not only expensive but also difficult to regenerate. In this regard, there is a growing interest among researchers in the last few years towards the use of adsorbents that are based and prepared from natural materials with an organic phase such as cellulose, hemicellulose, and lignin contents [7] which are marked by their efficiency and low-cost and their abundance in large quantities in developing countries such as Lgharb region in Morocco. Recently, quite a good number of research articles have been published on the utilization of low-cost adsorbents derived from biomass for wastewater treatment such as banana waste, moringa seed and lemon seed, straws, cotton, and palm fibers; rice (Oryza sativa) and coffee (Coffee Arabica) husk wastes were used [8–10].

Therefore, the present study aims to investigate the MB dye removal efficiency using cost-effective sustainable solid biomass sugarcane bagasse (SCB) adsorbent materials using batch and the close circuit under various physiochemical process conditions which may be applicable in actual large scale industrial treatment operation.

## 2. Materials and Methods

### 2.1. Materials

The materials are methylene blue C_16_H_18_ClN_3_S, sodium chloride (NaCl purity 99, 8%), chloride acid (HCl purity 37%), sodium hydroxide (NaOH purity 98%), and sugarcane bagasse (SCB).

### 2.2. Adsorbate

Methylene blue (MB) is an organic dye that is chosen for this study for its very high degree of purity (99%). It was used without any prior purification. Its characteristics are grouped in Table 1.

### 2.3. Adsorbent

Sugarcane bagasse is a natural waste abundantly available in El Ghareb region, Morocco. The sugarcane bagasse was collected from various local “sugarcane juice” venders; then, it was treated as follows: first, it was washed several times to remove the impurities, then dried in the oven at a temperature of 105°C for 24 h, and grind and sieved to a particle size equal to 250 *μ*m.

## 3. Characterization of SCB

This adsorbent's physical and chemical characteristics are viewed to possess high rates of efficiency of pollutants removal [11]. These characteristics are discussed in the following part.

### 3.1. Textural Characterization

The measurement of the specific surface of pore diameters and pore volume of SCB was obtained by using the nitrogen adsorption-desorption isotherm curve (Micromeritics ASAP 2010) using the Brunauer–Emmett–Teller (BET) method. Morphological analysis was made by scanning electron microscopy (SEM) type (VEGA3 TESCAN) coupled with EDX and quantitative analysis of the elemental composition of SCB was employed using X-ray energy dispersion spectroscopy (EDX).

### 3.2. Chemical Characterization

The chemical functions of the molecules present in the SCB were analyzed by Fourier Transform Infrared Spectroscopy (FTIR) type (Shimadzu, JASCO 4100). IR spectra were recorded over a wavelength range of 400 and 4000 cm^−1^. The zero point charge where pHzpc was determined to clarify the net charge was carried by the surface of the SCB.

### 3.3. Adsorption Procedure

The adsorption tests were conducted in the batch reactor, at ambient temperature; the colored synthetic solution of MB in the presence of the adsorbent SCB is stirred for an hour; and homogenization of the mixtures was carried out by a Shaker type incubator agitator (Jisico, model J-NSIL-R) with a stirring speed equal to 127 rpm. The adsorbate-adsorbent separation was performed using a 0.45 *μ*m diameter Wothman-type filtration system, and the supernatant absorbance was measured by a UV-Visible type spectrophotometer (Shimadzu 1601) at a wavelength corresponding to the maximum absorbance of MB (*λ*max = 665). Then, the residual dye concentration is determined from a calibration curve by Beer Lambert's law.

The adsorption capacity of MB by SCB is calculated by the following formula:

(1) $$\begin{array}{c} q_{t\;}=\frac{\left(C_{0}-C_{t}\right)*V}{m}, \end{array}$$

where *q*_*t*_ (mg·g^−1^) is the amount adsorbed at time *t* (min), *C*_0_ and *C*_*t*_ (mg·L^−1^) are the initial concentration and the concentration at time *t* in the dye, *V* (L) is the volume of the solution, and *m* (g) is the amount of the adsorbent in solution.

## 4. Results and Discussion

### 4.1. Adsorbent Characterization

#### 4.1.1. Nitrogen Adsorption-Desorption Isotherm

**Figure 2:**
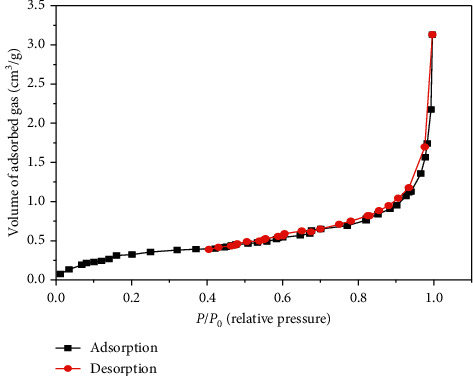
Nitrogen adsorption-desorption isotherm of the SCB.

The nitrogen adsorption-desorption isotherm is obtained at a temperature of 77.35 K after degassing at 80°C. The BET *S*_BET_ surface area was determined using the Brunauer–Emmett–Teller (BET) equation, the pore volume at saturation *V*_*P*_ is the volume of nitrogen corresponding to the highest relative pressure of *P*/*P*_0_ = 0.99, and the pore diameter *D*_*P*_ is calculated by the relation 4 *Vp*/*S*_BET_ [12]. The nitrogen adsorption-desorption isotherm obtained on sugarcane bagasse powder is shown in Figure 2.

According to the classification of isotherms (IUPAC), this curve has a sigmoidal shape, classified as type II, frequently found in fruits with a high sugar content [13], which means that the medium is nonporous or macroporous. The adsorption isotherm obtained shows that the SCB has a nonporous structure; this is confirmed by the textural measurements grouped in Table 2.

#### 4.1.2. Scanning Electron Microscopy (SEM)

**Figure 3:**
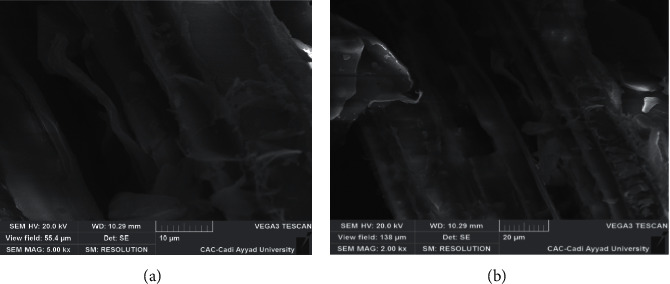
Observations of the structure of the SCB at the SEM. (a) Magnification 5.00 kx. (b) Magnification 2.00 kx.

The SEM images (Figure 3) illustrate the morphological structure of the SCB.

The observations obtained show that sugarcane bagasse has a fibrous structure, each elementary fiber has a compact structure that is aligned in the direction of the fiber axis, and sugarcane bagasse has a smooth and continuous surface [14, 15].

#### 4.1.3. Energy-Dispersive X-Ray Spectroscopy (EDX)

**Figure 4:**
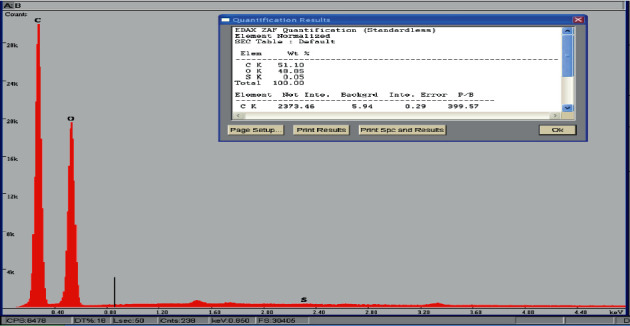
EDX spectrum of the SCB.

The surface elemental analysis of the SCB (Figure 4) indicates the presence of different chemical elements, and those results are grouped in Table 3. Which reveal the important presence of carbon and oxygen with a percentage of 51.10% and 48.85%, respectively, compared to the other chemical elements (S,...) which confirms the organic nature of our material [16], and the high oxygen content suggests that the surface of the adsorbent is more acidic.

### 4.2. Fourier Transform Infrared Spectroscopy (FTIR)

**Figure 5:**
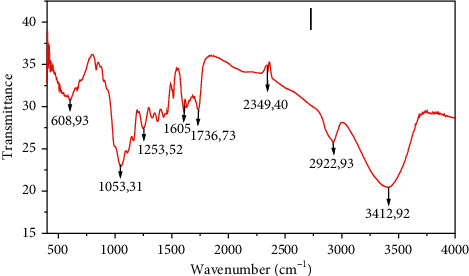
Infrared spectrum of the SCB.

The spectrum of sugarcane bagasse recorded by infrared spectroscopy between 400 and 4000 cm^−1^ is shown in Figure 5.

Figure 5 shows that the broadband at 3412.92 cm^−1^ is related to the elongation vibration of the O-H bond [17], mainly due to the presence of cellulose molecules [18]. The peak observed at 2922.93 is attributed to the C-H elongation and bending vibration of the CH_3_ methyl group [19]. The peak at 1736 cm^−1^ is due to the elongation of the carbonyl group of aldehydes and ketones [20]. The 1605 band is due to aromatic skeletal elongation vibrations existing in the lignin structure [21]. The 1053.31 cm^−1^ band is attributed to stretching vibrations in steric O=C-O-C compounds due to the existence of hemicellulose [22, 23], and the 608.93 cm^−1^ band corresponds to the bending modes of aromatic compounds [24].

### 4.3. Determination of Zero Point Charge (ZPC)

**Figure 6:**
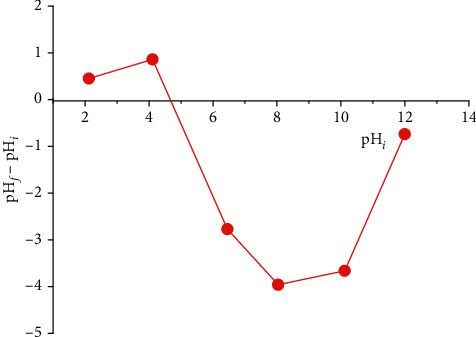
ZPC of the SCB.

Like many physiochemical variables such as pH, temperature, and ZPC, the latter is considered to be a significant factor that helps in determining the biosorption capacity of the biosorbent and the nature of binding sites [25, 26]. In this study, this method consists of preparing a series of 20 ml of a 5.10–2 M NaCl solution, after adjusting the initial pH (pH_*i*_) of each to values between 2 and 12, by the addition of HCl (0.1 M) or NaOH (0.1 M), a 0.2 g mass of the SCB is then added to the different solutions. All of this is left to stir at ambient temperature for 48 h until the final pH (pH_*f*_) has stabilized. The intersection of the curve ∆pH (pH_*f*_ − pH_*i*_) as a function of pH_*i*_ with the *x*-axis determines the ZPC (Figure 6).

The ZPC of the SCB is equal to 4.69, which expresses that the surface of the SCB is positively charged at a pH below 4.69 and negatively charged at a pH above 4.69. The low ZPC value also indicates the acidity of the adsorbent surface, which confirms the results of the elemental chemical composition which shows high oxygen content [26].

### 4.4. Effect of Reaction Parameters

#### 4.4.1. Effects of Adsorbent Amount

**Figure 7:**
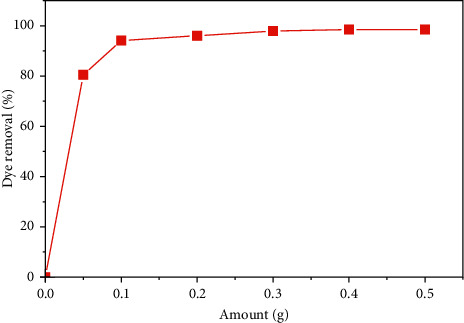
Effect of adsorbent amount on dye removal.

Biosorbent dosage has a major role in the dye removal process [26]. This study was conducted by varying the mass of the SCB between 0.05 and 0.5 g, keeping the other parameters constant: an ambient temperature, a solution pH of 6.4, an initial dye concentration of 25 mg·L^−1^, and a stirring speed of 127 rpm.  Figure 7 shows the effect of the adsorbent amount on dye removal.

According to the results obtained, the percentage of dye removal increases with the increase of the adsorbent amount from 80.27% for 0.05 g up to 98.49% for 0.5 g. This phenomenon is due to the increase of the specific surface area and the high availability of the adsorption sites [1]. For the continuation of the studies, we have chosen an amount equal to 0.2 g with an elimination percentage equal to 95.86%. The chosen amount minimizes the consumption of the amount of the SCB to half compared to 0.5 g with a percentage of 98.49% close to that of 0.2 g.

#### 4.4.2. Effect of Contact Time

**Figure 8:**
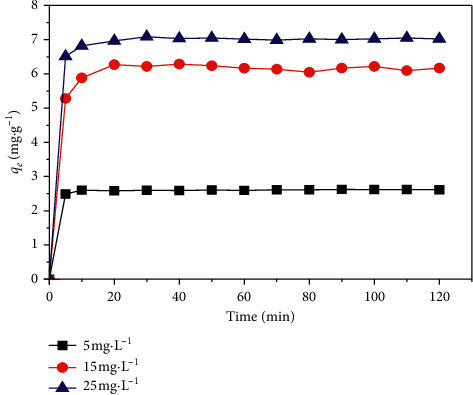
Effect of contact time on the adsorption capacity of MB.

The experiments were conducted in different contact times, and the variation in the amount of MB adsorbed by the SCB in the function of contact time (5–120 min) was observed as we set three initial concentrations of dye 5, 15, and 25 mg·L^−1^ as Figure 8 illustrates.

The results obtained show that the equilibrium time is independent of the initial dye concentration and that the amount of dye fixed on the adsorbent increases with the contact time. Figure 8 reveals the existence of two phases during the adsorption of the MB by the SCB: the first one is the fastest taking 5 minutes, which can be explained by the existence of a high affinity between the cations of the dye and the adsorbent [17], and the second is a slow phase where equilibrium is gradually reached after 20 minutes. This may be due to the saturation of the active sites in the support [27]. The contact time was set at 60 min for further studies.

#### 4.4.3. Effect of the Initial Dye Concentration

**Figure 9:**
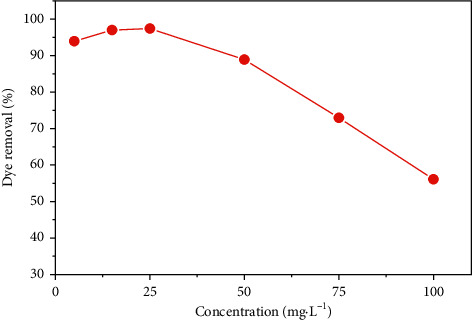
Percentage removal of MB variation with initial dye concentration by SCB.

The effect of the initial concentration of MB on its retention rate by the SCB was studied at different initial concentrations, ranging from 5 to 100 mg·L^−1^, and a constant mass of the SCB of 0.2 g at ambient temperature for 60 minutes (Figure 9).

Figure 9 shows that the percentage of MB removal increases for low concentrations, up to a maximum obtained at 97% for a concentration of 25 mg·L^−1^. This can be explained by the availability of active sites which are much higher than the amount of dye introduced. However, at high concentrations, the percentage decreases from 97% to 56% due to a lack of available active sites.

#### 4.4.4. Effect of pH

**Figure 10:**
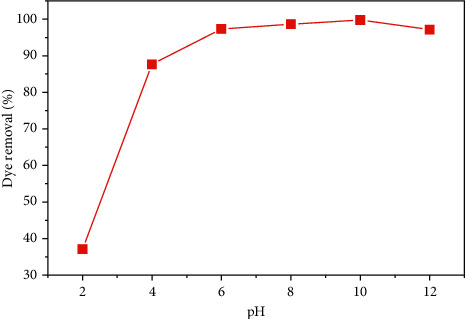
Effect of pH solution on dye removal.

The solution pH plays a significant role in the sorption process as it can affect the surface charge of the adsorbent and the molecular state of the dye molecule. In other words, it interrupts both the solution's chemistry of dyes and functional groups of the adsorbents. Meanwhile, it seems that the adsorption capacity of the dye depends on the pH of the solution [25, 28]. The variation of biosorption capacity of SCB for the removal of MB dye was studied in the pH range of 2 to 12 by adding HCl (chloride acid) purity 37% (0.1 M) or NaOH (sodium hydroxide) purity 98% (0.1 M), keeping other variables constant (0.2 g/50 ml biosorbent dose, 25°C temperature, 127 rpm, and 60 min contact time).  Figure 10 shows the effect of pH solution on dye removal.

The results indicate that the basic pH was favorable for the removal of MB. The optimum pH for maximum dye removal of SCB was found to be 10, (dye removal = 99.30%).

#### 4.4.5. Effect of Ionic Strength

**Figure 11:**
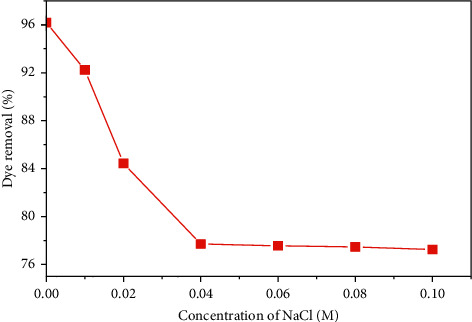
Effect of concentration of NaCl on the removal dye.

Wastewater contains various elements such as salts, organic, and metal ions. The presence of those ions is the reason for a high ionic strength which affects the performance of the adsorption process significantly [29]. The study of the influence of this parameter on the adsorption phenomenon is carried out by adding variable amounts of NaCl (sodium chloride, purity 99.8%) of concentration ranging from 0 to 0.1 M to solutions of the dye of initial concentration of 25 mg·L^−1^ and a mass of 0.2 g of the adsorbent. The influence of the initial concentration of NaCl on the rate of removal of MB by SCB is shown in Figure 11.

According to these results, we notice a great decrease in the rate of elimination of the dye with the increase of the NaCl concentration from 93.63% to 77.71%. Above a concentration of 0.04 M NaCl, there is always a decrease but at a very low rate. This phenomenon can be attributed to the fact that Na + ions accumulate in greater numbers next to the surface and thus screen the fixation sites [30].

#### 4.4.6. Effect of Temperature

**Figure 12:**
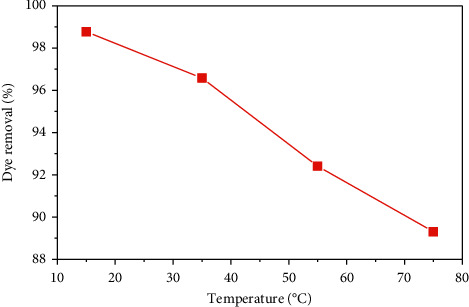
Effect of temperature on MB adsorption by the SCB.

The influence of the temperature on the adsorption phenomenon was carried out by adding 0.2 g/50 ml of SCB to a solution of MB at a concentration of 25 mg/L, at a temperature varying between 15 and 75°C (Figure 12).

Figure 11 shows that the MB removal rate on the SCB decreases from 98.75% to 89.22% with increasing temperature from 15 to 75°C. This decrease can be explained by the destruction of the adsorption sites [31], which means that the increase in temperature adversely affects the adsorption mechanism, so the reaction is exothermic in nature.

### 4.5. Kinetics Study

MB adsorption kinetics data from the SCB are modeled by three models: pseudo-first-order, pseudo-second-order, and intraparticle diffusion.

The pseudo-first-order kinetic model, evaluated by the Lagergren relation [32], is described by the following equation:

(2) $$\begin{array}{c} \frac{\text{d}q_{t}}{\text{d}t}=K_{1}\left(q_{e}-q_{t}\right). \end{array}$$

After the integration for the boundary conditions *q*_*t*_ = 0 to *t* = 0 and *q*_*t*_ = *q_t_* to *t* = *t*, the equation becomes

(3) $$\begin{array}{c} \ln\left(q_{e}-q_{t}\right)=\ln\;q_{e}-K_{1}t. \end{array}$$

The quantity adsorbed at equilibrium *q*_*e*_ and the velocity constant *k*_1_ are obtained from the intercept and the slope of the curve ln (*q*_*e*_ − *q*_*t*_) versus time *t*, respectively.

The application of Blanchard's model allows us to define the pseudo-second-order of the reaction in a sorption process [33]:

(4) $$\begin{array}{c} \frac{\text{d}q_{t}}{{\text{d}}_{t}}=K_{2}{\left(q_{e}-q_{t}\right)}^{2}. \end{array}$$

The integration of the equation for the boundary conditions *q*_*t*_ = 0 at *t* = 0 and *q*_*t*_ = *q*_*t*_ at *t* = *t* gives

(5) $$\begin{array}{c} \frac{t}{q_{t}}=\frac{1}{K_{2}\times q_{e}^{2}}+\frac{t}{q_{e}}. \end{array}$$

The quantity adsorbed at equilibrium *q*_*e*_ and the velocity constant *K*_2_ are determined, respectively, from the slope and the intercept at the origin of the *t*/*q*_*t*_ curve as a function of time *t*.

The intraparticle diffusion (internal transport) is also used to identify the diffusion mechanism. It is presented by the following equation:

(6) $$\begin{array}{c} q_{t}=K_{i\;d}\cdot t^{1/2}+C. \end{array}$$

By tracing *q*_*t*_ as a function of *t*^1/2^, the constants *K*_*id*_ and *C* are deduced from the slope and intercept, respectively.

The best model that describes adsorption kinetics is the one with the highest *R*^2^ linear regression coefficient.

**Figure 13:**
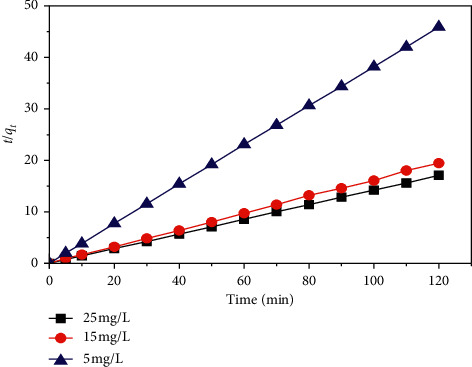
Pseudo-second-order model of adsorption kinetics.

Figure 13 presents the *t*/*q*_*t*_ curve in the function of time *t* at different initial MB concentrations of the pseudo-second-order model for MB adsorption by the SCB and shows improved linearity.

Table 4 summarizes the results of the three models of MB adsorption kinetics by the SCB. According to this table, the adsorption kinetics is described perfectly with the pseudo-second-order model and the intraparticle diffusion model, with a correlation coefficient which is equal to 1 for the pseudo-second-order kinetic model and 0.96 for the intraparticle diffusion model; the calculated value of the adsorbed quantity at the equilibrium *q*_*e*,cal_ is very close to the experimental value *q*_*e*_,_exp_. On the other hand, the pseudo-first-order model represents a poor correlation. The velocity constant *K*_2_ shows that the retention of MB by the SCB is quite fast [27].

### 4.6. Adsorption Isotherms

Some of the important roles of the adsorption isotherms are that they allow the description of how the adsorbate interacts with the adsorbent, illustrate the type of accumulation of adsorbate on the adsorbent, and analyze the adsorption equilibrium [34]. Experimental data for the MB adsorption isotherms in the SCB were modeled using two models: the Langmuir model and the Freundlich model illustrated below.

### 4.7. Langmuir Model

Langmuir's model supposes that the adsorption comes from the monolayer coverage of the adsorbate on a homogeneous surface; that is, once a dye molecule takes up at a site, no further adsorption can take place at that site [35], and it is illustrated that

(7) $$\begin{array}{c} q_{e}=\frac{K_{e}q_{m}C_{e}}{1+K_{L}C_{e}}. \end{array}$$

The linear transform of this model has the following equation [36]:

(8) $$\begin{array}{c} \frac{C_{e}}{q_{e}}=\frac{1}{q_{m}\times K_{L}}+\frac{C_{e}}{q_{m}}. \end{array}$$

With *q*_*e*_ and *q*_*m*_ (mg·g^−1^) being the amount adsorbed at equilibrium and the maximum amount adsorbed at saturation of the monolayer, *C*_*e*_ (mg·L^−1^) is the equilibrium concentration and *K*_*L*_ (L·mg^−1^) is the Langmuir constant.

By tracing *C*_*e*_/*q*_*e*_ according to *C*_*e*_, the Langmuir model is checked if a straight line of slope 1/*q*_*m*_ and ordinate at the origin 1/*q*_*m*_*K*_*L*_ is obtained.

We can check whether adsorption is favorable or not by the equilibrium parameter *R*_*L*_ given as

(9) $$\begin{array}{c} R_{L}=\frac{1}{1+K_{L}C_{0}}. \end{array}$$

The adsorption is irreversible (*R*_*L*_ = 0), favorable (0 < *R*_*L*_ < 1), linear (*R*_*L*_ = 1), and unfavorable (*R*_*L*_ > 1) [37].

### 4.8. Freundlich Model

Freundlich's model is based on an empirical equation used to model adsorption isotherms on energetically heterogeneous surfaces [38]. It is expressed by the following relationship [39]:

(10) $$\begin{array}{c} q_{e}=K_{F}C_{e}^{1/n}, \end{array}$$

where *q*_*e*_ (mg·g^−1^) is the equilibrium amount adsorbed, *K*_*F*_ and *n* are Freundlich's constants, and *C*_*e*_ (mg·L^−1^) is the equilibrium concentration of the solute.

The logarithmic model of this relationship makes it possible to verify its linear transformation [36]:

(11) $$\begin{array}{c} \ln\;q_{e}=\frac{1}{n}\ln\;C_{e}+\ln\;K_{F}. \end{array}$$

The values of *K*_*F*_ and *n* are determined experimentally by drawing ln  *q*_*e*_ according to ln *C*_*e*_.

**Figure 14:**
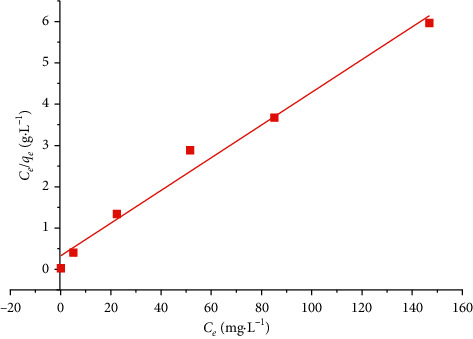
Langmuir model for MB adsorption by SCB at 25°C.

**Figure 15:**
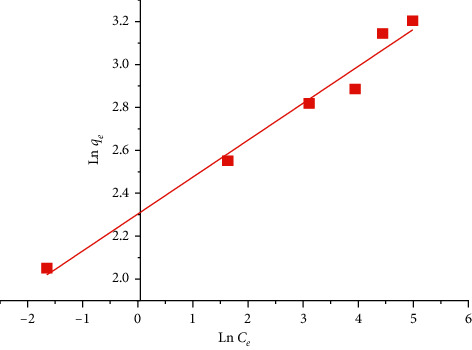
Freundlich model for MB adsorption by the SCB at 25°C.

The isotherms of MB adsorption by the SCB at 25°C are presented according to the Langmuir model (Figure 14) and the Freundlich model (Figure 15).

Table 5 presents the values of the adsorption equilibrium parameters according to the Langmuir and Freundlich model.

According to these results, the correlation coefficient of the Langmuir model is equal to 0.98, as it is close to the Freundlich correlation coefficient of 0.97. This means that the process of MB adsorption by SCB is perfectly described by both Langmuir and Freundlich models and the separation factor *R*_*L*_ < 1 indicates that the adsorption is favorable.

### 4.9. Thermodynamic Study

Thermodynamic parameters such as enthalpy Δ*H*°, entropy Δ*S*°, and free enthalpy Δ*G*° were calculated at different temperatures of 15, 35, 55, and 75°C to describe the reaction of MB adsorption by the SCB from the following equations [40]:

(12) $$\begin{array}{c} Ln\;K_{d}=\frac{\Delta S^{{}^{\circ}}}{R}-\frac{\Delta H^{0}}{RT},\\ \Delta G^{0}=\Delta H^{0}-T\Delta S^{0}. \end{array}$$

*K*_*d*_=*q*_*e*_/*C*_*e*_ is the distribution coefficient; *q*_*e*_ (mg·g^−1^) is the amount adsorbed at equilibrium; *R* is the perfect gas constant and *T* (K) is the temperature of the solution; and *C*_*e*_ (mg·L^−1^) is the equilibrium concentration.

The thermodynamic parameters play a significant role as they provide information about the spontaneity and endo- or exothermicity of the adsorption process and the increment or decrease of randomness at the solid-liquid interface [41].

**Figure 16:**
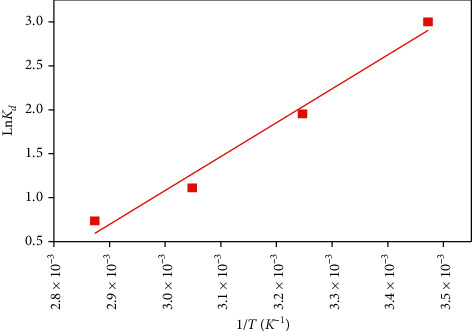
Modelling of the thermodynamic study.

The plot of ln  *K*_*d*_ as a function of 1/*T* (Figure 16) gives a straight line of slope-Δ*H*°/*R* and an intercept at the origin Δ*S*°/R. The results obtained are grouped in Table 6.

These results show that the adsorption process of MB on the SCB is exothermic in nature and can be qualified as physical adsorption since the value of Δ*H*° is negative and greater than −40 kJ·mol^−1^; the negative value of Δ*S*° indicates that the molecules of the dye are more organized at the solid/liquid interface than in solution, hence a decrease in molecular disorder. Negative values of Δ*G*° indicate that adsorption is spontaneous and the increase in these values as the temperature increases from 15 to 75°C shows that the feasibility of adsorption decreases at elevated temperatures [42].

## 5. Conclusion

The adsorption process of MB on the SCB was the aim of this study. The results obtained show that the removal rate of MB increases from 80.27% to 98.49% with the increase of the mass of adsorbent from 0.05 g to 0.5 g due to the increase in specific surface area. The maximum removal of dye was observed at pH 10 and the biosorption process has reached equilibrium at 60 min. The contact time effect indicated that the equilibrium time is independent of the initial MB concentration, adsorption is very fast after 5 min, and equilibrium time is reached at 20 min. On the other hand, the rate of elimination decreases with increasing temperature. Therefore, an exothermic reaction occurs.

The kinetic and isothermal studies of the adsorption mechanism are perfectly described by pseudo-second-order kinetics and both Langmuir and Freundlich models as they perfectly present the adsorption of MB on the SCB. Finally, the thermodynamic studies have shown that the adsorption of MB by the SCB is exothermic, feasible, and spontaneous.

To conclude, SCB is found to be a good biosorbent for MB removal which makes it a great alternative for wastewater and dye effluents treatment, especially today, as we need to protect the environment using environment-friendly processes like this.

## Figures and Tables

**Table 1:** Chemical characterization of MB.

Dye	Methylene blue
Structure	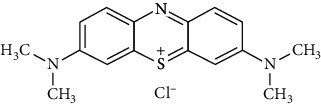
Molecular formula	C_16_H_18_ClN_3_S
Molecular weight (g/mol)	319.85
Maximum wavelength *λ*max (nm)	665

**Table 2:** Results of the analyses of the porous network by nitrogen adsorption.

Sample	Pore diameter (Å)	Specific surface (m^2^/g)	Pore volume (cm^3^/g)
Sugarcane bagasse	57.285	0.7340	0.000679

**Table 3:** Chemical composition of the SCB adsorbent.

Element	C (carbon)	O (oxygen)	S (sulfur)
Mass percentage	51.10	48.85	0 .05

**Table 4:** Kinetic parameters related to the adsorption of MB on the SCB.

	Dye concentration (mg·L^−1^)	5	15	25
	*q*_*e*,exp_ (mg·g^−1^)	2.621	6.285	7.047

Pseudo-first-order kinetic model	*k*_1_ (min^−1^)	2.1591	1.4277	1.1817
	*q*_*e*,cal_ (mg·g^−1^)	0.968	0.991	0.9814
	*R* ^2^	0.66	0.092	0.55

Pseudo-second-order kinetic model	*k*_2_ (g·mg^−1^·min^−1^)	2.329	10.156	0.991
	*q*_*e*,cal_ (mg·g^−1^)	2.621	6.153	7.032
	*R* ^2^	1	0.997	1

Intraparticle diffusion model	*K*_*id*1_ (g·mg^−1^·min^−1/2^)	0.8769	1.9524	2.2958
	*C* (mg·g^−1^)	0.12	0.2071	0.3122
	*R* ^2^	0.94	0.96	0.93
	*K*_*id*2_ (g·mg^−1^·min^−1/2^)	0.0061	0.1168	0.1142
	*C* (mg·g^−1^)	2.5538	5.5959	6.4559
	*R* ^2^	0.76	0.69	0.99

**Table 5:** Adsorption parameters of the MB on the SCB according to the Langmuir and Freundlich models.

	Parameters	Values
Langmuir model	*K*_*L*_ (L·mg^−1^)	0.1225
	*q*_max_ (mg·g^−1^)	49.261
	*R*_*L*_	0.246
	*R* ^2^	0.98

Freundlich model	*K*_*F*_	10.0071
	1/*n*	0.172
	*R* ^2^	0.97

**Table 6:** Thermodynamic parameters related to the adsorption of the MB dye on the SCB.

Thermodynamic parameters	Temperature (°K)			
	288	308	328	348
Δ*G*° (kJ·mol^−1^)	−6.87	−5.19	−3.51	−1.83
Δ*S*° (kJ·mol^−1^·K^−1^)	−0.084			
Δ*H*° (kJ·mol^−1^)	−31.062			

## Data Availability

The datasets generated and/or analyzed during the current study are available from the corresponding author on reasonable request.
